# Genetics of resistance to trimethoprim in cotrimoxazole resistant uropathogenic *Escherichia coli*: integrons, transposons, and single gene cassettes

**DOI:** 10.3389/fmicb.2024.1395953

**Published:** 2024-06-12

**Authors:** María Eloísa Poey, Eliana de los Santos, Diego Aznarez, César X. García-Laviña, Magela Laviña

**Affiliations:** ^1^Sección Fisiología & Genética Bacterianas, Facultad de Ciencias, Montevideo, Uruguay; ^2^Sección Bioquímica, Facultad de Ciencias, Montevideo, Uruguay

**Keywords:** antibiotic resistance, trimethoprim, cotrimoxazole, integrons, gene cassettes, transposons, plasmid transfer, *Escherichia coli*

## Abstract

Cotrimoxazole, the combined formulation of sulfamethoxazole and trimethoprim, is one of the treatments of choice for several infectious diseases, particularly urinary tract infections. Both components of cotrimoxazole are synthetic antimicrobial drugs, and their combination was introduced into medical therapeutics about half a century ago. In Gram-negative bacteria, resistance to cotrimoxazole is widespread, being based on the acquisition of genes from the auxiliary genome that confer resistance to each of its antibacterial components. Starting from previous knowledge on the genotype of resistance to sulfamethoxazole in a collection of cotrimoxazole resistant uropathogenic *Escherichia coli* strains, this work focused on the identification of the genetic bases of the trimethoprim resistance of these same strains. Molecular techniques employed included PCR and Sanger sequencing of specific amplicons, conjugation experiments and NGS sequencing of the transferred plasmids. Mobile genetic elements conferring the trimethoprim resistance phenotype were identified and included integrons, transposons and single gene cassettes. Therefore, strains exhibited several ways to jointly resist both antibiotics, implying different levels of genetic linkage between genes conferring resistance to sulfamethoxazole (*sul*) and trimethoprim (*dfrA*). Two structures were particularly interesting because they represented a highly cohesive arrangements ensuring cotrimoxazole resistance. They both carried a single gene cassette, *dfrA14* or *dfrA1*, integrated in two different points of a conserved cluster *sul2-strA-strB*, carried on transferable plasmids. The results suggest that the pressure exerted by cotrimoxazole on bacteria of our environment is still promoting the evolution toward increasingly compact gene arrangements, carried by mobile genetic elements that move them in the genome and also transfer them horizontally among bacteria.

## 1 Introduction

Cotrimoxazole (SXT), the combination of trimethoprim (TMP) and sulfamethoxazole (SMX), is widely used in the treatment of several infections, particularly those affecting the urinary tract. As an effective and inexpensive medication, it is on the list of the World Health Organization of essential medicines (World Health Organization, 2023). Its antibiotic components act as competitive inhibitors of two enzymes of the folic acid (Fol) synthesis pathway, and their action is enhanced when combined. Due to their mode of action, they are generically called antifolates. SMX belongs to the family of sulfonamides, which includes several compounds that inhibit dihydropteroate synthase, the first enzyme in the Fol pathway, and TMP inhibits dihydrofolate reductase, the third and last enzyme required to produce tetrahydrofolate. This latter compound is the active cofactor that performs the essential function of providing one-carbon units to many biosynthetic reactions in cells (Green and Matthews, 2007). Sulfonamides opened the antibiotic era, being the first antibacterial drugs used systemically in medicine, since the 1930s. TMP was developed and employed in the 1960s, and SXT came into use shortly after due to the observed improved effect of the combination of TMP and SMX (Huovinen, 1997).

Over time, resistance to SXT emerged and has steadily increased, limiting its clinical use. Resistance to this combination is due to the sum of resistance to both its components, and is particularly frequent among Gram-negative bacteria. It is mediated by genes belonging to the accessory gene pool encoding variants of the dihydropteroate synthase and dihydrofolate reductase that are resistant to SMX and TMP, respectively. Currently, four SMX resistance genes have been described (*sul1, 2, 3*, and *4*) and several dozen for TMP resistance (*dfrA* numbered in the order in which they were described, and a *dfrB* series of eight variants) (Sköld, 2001; Alonso and Gready, 2006; Ambrose and Hall, 2021; de los Santos et al., 2021; Alcock et al., 2023).

In principle, Gram-negative strains resistant to SXT contain at least one *sul* gene and one *dfr* gene. These are usually associated with mobile genetic elements that enable them to move within the bacterial genome, such as integrons and transposons, and to be transferred horizontally between bacteria, typically being carried on conjugative plasmids. The *sul* and *dfrA* genes are significantly associated with class 1 and 2 integrons. These are genetic elements often referred to as “clinical integrons” because they confer resistance to multiple antibiotics and are commonly found in Gram-negative clinical isolates. These features distinguish them from other integrons found in environmental bacteria (Labbate et al., 2009).

Integrons consist of a conserved platform and a variable region. In the platform they encode a site-specific recombination system that captures and integrates free gene cassettes into an attachment site (*attI*) so that they become part of the integron and are expressed as an operon from a promoter also provided by the platform. Then, the variable region of an integron is the array of gene cassettes it contains. As free circular molecules, gene cassettes usually contain a single gene without a promoter, followed by its specific attachment site (*attC*). The integrase recombines the *attC* and the *attI* sites to operate the gene cassette integration. The mechanism described above implies that the first cassette found in an array was in fact the last one to be integrated. Also, since it is located immediately after the promoter, this cassette would be the one expressed at a higher level (Hall, 2012; Cury et al., 2016).

**Figure 1:**
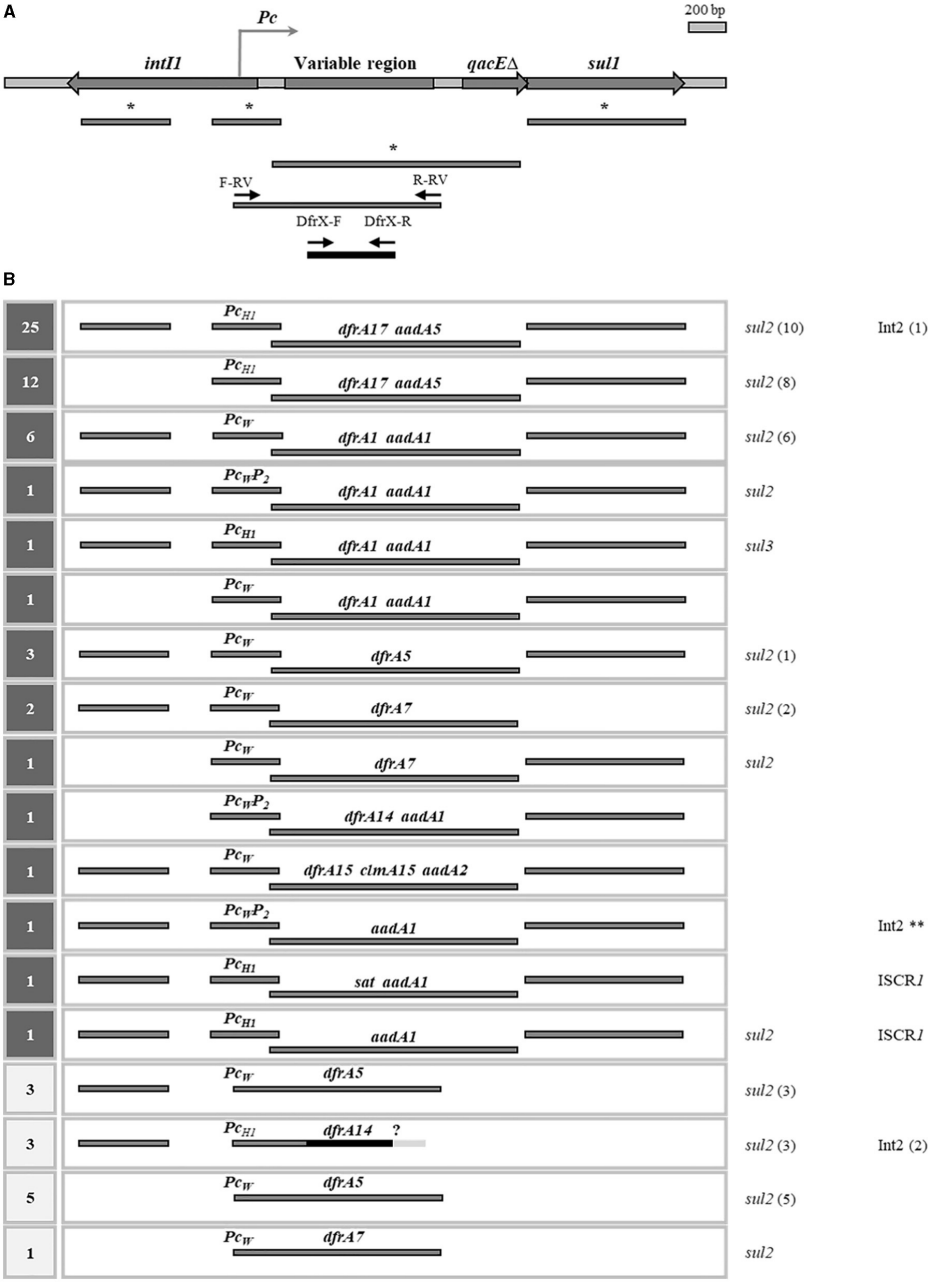
Class 1 integrons or its remnants in the cotrimoxazole resistant strains. **(A)** General structure of an Int1: genes are indicated with thick arrows; the genetic content of the variable region is not specified due to its variability. Below, bars indicate the generated amplicons to detect different parts of Int1 in previous (*) and present determinations. Small arrows, new primers used in this work. **(B)** Int1 structures detected in the 69 strains with Int1 sequences. At left, number of strains carrying each type of structure: in dark gray squares, previously determined; in clear squares, determined in this work. At right, presence of *sul2, sul3*, Int2, and ISCR*1*, with the number of strains in brackets. ?, unknown genetic content in the right end of the variable region. **, TMP^R^ is encoded by the *dfrA1* gene from the Int2. Previous determinations were described in Poey and Laviña (2014, 2018) and de los Santos et al. (2021).

Class 1 integrons (Int1) are most prevalent in clinical isolates of Enterobacteriaceae and other Gram-negative families. They usually contain one or two genes in their variable region: a *dfrA* gene and/or an *aadA* gene, conferring TMP and streptomycin resistance, respectively. The presence of other gene cassettes has been repeatedly reported, but is less frequent (Domingues et al., 2015). In the Int1, the platform is divided by the variable region into two parts: (i) a 5′ conserved segment (5′ CS), containing the site-specific integrase gene *intI1*, the *Pc* promoter that directs transcription of the integrated gene cassettes, and the attachment site *attI*; and (ii) a 3′ conserved segment (3′ CS), containing a truncated gene for an export pump (*qacE*Δ) and the *sul1* gene, conferring resistance to SMX (Figure 1A) (Labbate et al., 2009). As to the *Pc* promoter, several variants with different strengths have been recognized, being most frequent *Pc*_*W*_ (weak) and *Pc*_*H*1_ (hybrid 1). In addition, some integrons contain a second promoter downstream of *Pc*, called *P*_2_, which usually accompanies the *Pc*_*W*_ variant, enhancing its strength (*Pc*_*W*_*-P*_2_) (Poey and Laviña, 2014). It is worth mentioning that *sul1*, which is not a gene cassette, is the only resistance gene residing in the conserved platform of Int1s (Labbate et al., 2009). In a previous study we showed that even when *sul1* is deleted, Int1^+^ strains remain resistant to SMX thanks to the presence of an unlinked *sul2* gene or, less frequently, of a *sul3* gene. This led us to propose that the presence of an SMX resistance gene would be a requirement for Int1^+^ strains (de los Santos et al., 2021). Therefore, in principle, every strain bearing an Int1 contains at least one *sul* gene, and in the vast majority, a *dfrA* gene in the variable region. The result is that all Int1^+^ strains are SMX resistant and most of them are also SXT resistant.

Class 2 integrons (Int2) have a structure quite similar to that of Int1, but lack the 3′segment of the platform and have a nonsense mutation that inactivates the integrase gene *intI2*. Therefore, its structure is fixed and, in principle, its variable region is no longer variable. Nevertheless, it appears that the Int1 integrase would be able to trans-act on the Int2 attachment site, generating some variability; in fact, it is not uncommon to find both integrons 1 and 2 in the same strain. In its classical fixed structure, Int2 carries three antibiotic resistance genes in the form of gene cassettes: *dfrA1, sat2*, and *aadA1*, conferring resistance to TMP, streptothricin and streptomycin, respectively. This integron exemplifies very well the association of these types of elements with transposons. Int2 is located inside Tn7, so it moves while being carried by this transposon. Regarding its relationship with the resistance to antifolates, Int2 contains the *dfrA1* gene cassette, and frequently resides in bacteria containing the *sul2* gene elsewhere (Deng et al., 2015; de los Santos et al., 2021). Then, most Int2^+^ strains have a SXT resistant phenotype.

**Figure 2:**
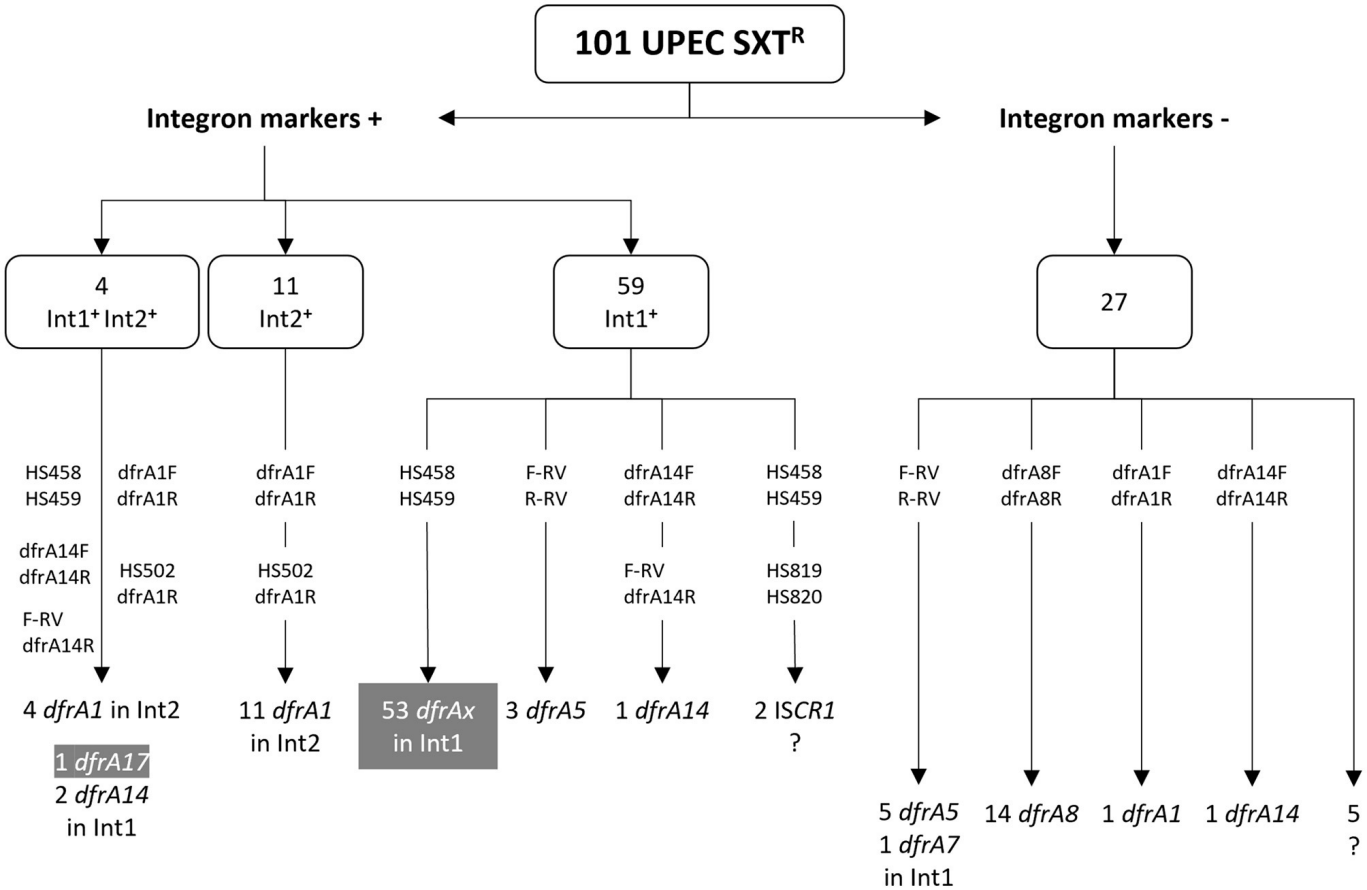
PCR amplifications for the detection of *dfrA* genes in the cotrimoxazole resistant strains. The number of strains with or without integron markers is shown above. Attached to vertical arrows, primer pairs that gave rise to amplicons. Below, type and number of *dfrA* genes identified; dfrAx generically designates several types of dfrA genes. In gray boxes, *dfrA* genes previously identified in amplicons with primer pair HS458 and HS459 (Poey and Laviña, 2018; de los Santos et al., 2021). ?, strains in which the gene responsible for the TMP^R^ phenotype was not found.

In this communication we address the genetic basis of cotrimoxazole resistance in a collection of 101 uropathogenic *E. coli* (UPEC) strains, gathering present with previous results (Poey and Laviña, 2014, 2018; de los Santos et al., 2021). These strains belong to a broader collection of 230 clinical isolates which had been gathered following an epidemiological design conducted in 2007–2009 in a hospital in Montevideo, Uruguay (Poey et al., 2012). Therefore, this collection is representative of the UPEC population circulating at that place and time. Since then, it has been studied from different points of view, including antibiotic resistance, presence of *sul* genes and of clinical integrons. Regarding antifolate resistance, more than half of the strains are resistant to one or both SMX and TMP, with a clear predominance of SXT resistance. These latter strains are 101, being the subject of interest of the present study. As to the genetic basis of their SXT^R^ phenotype, they all contain at least one type of *sul* gene: *sul1* (*n* = 27), *sul2* (*n* = 46), *sul1* and *sul2* (*n* = 27), and *sul1* and *sul3* (*n* = 1), what explains their SMX resistance. Regarding the basis of their TMP^R^, we only had information on 54 strains, specifically those that contain an Int1 whose variable region could be amplified and sequenced, and which proved to have a *dfrA* gene cassette. These are: *dfrA17* (*n* = 37), *dfrA1* (*n* = 9), *dfrA5* (*n* = 3), *dfrA7* (*n* = 3), *dfrA14* (*n* = 1), and *dfrA15* (*n* = 1). In addition, the *Pc* promoter is also identified, thus completing the information on the central expression unit in these Int1s (Figures 1, 2) (Poey and Laviña, 2014, 2018; de los Santos et al., 2021). Therefore, the genetic basis of the SXT^R^ of 54 strains had been previously elucidated and their TMP^R^ proved to be due to *dfrA* gene cassettes included in class 1 integrons. In this work we devoted to complete the analysis of the genetic bases underlying TMP^R^ in the 47 remaining SXT^R^ strains and succeeded in identifying them in 40. Many were associated with Int1 remnants or with Int2, while others were found in other apparently less frequent genetic structures, including a novel gene arrangement.

## 2 Materials and methods

### 2.1 Bacterial strains and culture conditions

One hundred and one STX^R^ UPEC isolates were considered for the analysis: 28 were isolated from pregnant women (PW) and 73 from children (CH). They belonged to a larger UPEC collection of 230 isolates from the Laboratory of Bacteriology of an obstetric and pediatric hospital in Montevideo, Uruguay (Centro Hospitalario Pereira Rossell). The isolates were collected in 2007 (PW) and 2008–2009 (CH) using the following inclusion criteria: they were all the *E. coli* isolates proceeding from different patients with urinary tract infection or that proved to be different when coming from the same patient. The 230 isolates were previously characterized for their phylogeny, virulence, antibiotic resistance and for the presence of class 1, 2, and 3 integrons, and were then considered as strains. Of the 101 SXT^R^ strains, 63 contained an Int1 (by the criterion of being *intI*1^+^ and/or *sul*1^+^), 15 an Int2 (by the criterion of being *intI*2^+^), and four of them had both Int1 and Int2, and none had a class 3 integron (Poey et al., 2012; Poey and Laviña, 2014; de los Santos et al., 2021).

Strains were grown in LB medium at 37°C. Antibiotics were added to media at the following final concentrations: SMX, 800 μg/ml; TMP, 100 μg/ml; SXT, the combination of TMP, 100 μg/ml plus SMX, 500 μg/ml; nalidixic acid (NAL), 40 μg/ml, and kanamycin (KAN), 30 μg/ml.

### 2.2 PCR assays

The presence of genes encoding TMP resistance was searched for by PCR and primers designed are presented in Table 1. Primer pairs were used to amplify internal segments of some selected *dfrA* genes. Since our study was being performed on *E. coli* strains, the most common *dfrA* variants for this species were surveyed in the GenBank using the Blastn program (https://blast.ncbi.nlm.nih.gov/Blast.cgi) and, as queries, the *dfrA* sequences in CARD (Comprehensive Antibiotic Resistance Database) (https://card.mcmaster.ca/ontology/40753; Alcock et al., 2023). Nine *dfrA* variants were selected because they appeared in more than 15 releases as of June 2020. These genes and their frequencies of occurrence were: *dfrA17* (461), *dfrA12* (274), *dfrA1* (165), *dfrA7* (144), *dfrA14* (111), *dfrA5* (70), *dfrA27* (41), *dfrA15* (23), and *dfrA8* (17). The presence of the uncommon genes *dfrB* (8 variants) was assayed with a generic pair of primers. In addition, primers F-RV and R-RV were designed to generate amplicons tightly adjusted to the *Pc*-gene cassettes region, which proved to be particularly useful when the Int1 was deleted for most of the 3'CS (Figure 1). Complex class 1 integrons were surveyed through the detection of the ISCR*1* (Insertion Sequence Common Region associated to Int1) with primers described previously: HS819 (5′ GGGCCAGGTCTTGAGTATCG 3′) and HS820 (5′GCTTCGGCCATCACACC 3′), using an annealing temperature of 55°C and generating an expected product size of 522 bp (Márquez et al., 2008). The linkage between the *intI2* and the *dfrA1* genes in Int2^+^ strains was assayed with primers dfrA1-15R and HS502 (5′ GTAGCAAACGAGTGACGAAATG 3′) (Márquez et al., 2008), with an annealing temperature of 52°C and an expected product size of 1,619 bp.

**Table 1:** Primers designed and used for PCR amplifications.

**Gene (product size in bp)**	**Primer name**	**Primer sequence 5^′^> 3^′^**	**Anneal. temp. (°C)**
*dfrA1* (283)	dfrA1F	CACGTTCAAGTTTTACATCTGAC	55
	dfrA1-15R	CCCTTTTGCCAGATTTGGTAACTAT	
*dfrA5* (180)	dfrA5F	TGGACGGCCGATAATGACAAC	59
	dfrA5R	CATCTCCTTCCGGCTCAATATC	
*dfrA7* (294)	dfrA7F	GGTCAGCAAAAGGTGAGCAGT	55
	dfrA7-17R	CAACGTGAACAGTAGACAAATG	
*dfrA8* (296)	dfrA8F	ATGATCGAGCTTCATGCCATT	54
	dfrA8RE	ACGCTCTCTTCTTGAGCGAACC	
*dfrA12* (480)	dfrA12F	TGAACTCGGAATCAGTACGCATT	57
	dfrA12R	ATAAACGGAGTGGGTGTACGGAA	
*dfrA14* (370)	dfrA14F	CATTGATGGCTGCGAAAGCGAA	55
	dfrA14R	AAAACATCCCCCTCTGGCTC	
*dfrA15* (285)	dfrA15F	AACTCGTTCAAGCTTCACTTCCAG	59
	dfrA1-15R	See above	
*dfrA17* (294)	dfrA17F	GGTCAGTAAAAGGTGAGCAAC	55
	dfrA7-17R	See above	
*dfrA27* (400)	dfrA27F	AGCAAGAAATGGGGTTATTGG	54
	dfrA27R	TTCCTGCTCGAACACCACATT	
*strA* (857)	strAF	CCTCCGCGCTTCATCAGAAAACT	60
	strAR	AACAGGCGGCATGAACATCAACCC	
*dfrB* (133)	dfrBF	TGGGAGATCGCGTGCGCAAGAA	60
	dfrBR	GGATAAATCTGTACTGAGCCTGGGTG	
*folA* (766)	folAF	GAACCGGAAACGAAACCCTCAT	61
	folAR	GATATAGGAAGGCCGGATAAGAC	
Int1 *Pc* and variable reg.	F-RV	AAACGGATGAAGGCACGAACCCAGT	58
	R-RV	GGCTGTGAGCAATTATGTGCTTAGT	

PCR-amplifications were performed in a volume of 30 μl containing 1X buffer, 200 μM of each deoxynucleotide triphosphate, 200 nM of each primer, 2U of High Taq polymerase (BIORON), and 10 μl of cell lysate (prepared by the boil lysis method). The conditions used were: 2 min at 94°C, followed by 30 cycles of 94°C for 30 s, the annealing temperature for 30 s and extension at 72°C for 30 s or more, depending on the size of the amplicon, and a final extension step at 72°C for 2 min.

### 2.3 Conjugation and transformation experiments

Conjugation was performed essentially as described previously (Poey and Laviña, 2018). Several UPEC SXT^R^ and sensitive to KAN and NAL were used as donors and an *E. coli* K12 derivative strain (*hsdR::kan gyrA*; KAN^R^ and NAL^R^) as recipient. Transconjugants were selected on LB plates supplemented with TMP and KAN. In parallel, cultures of the parental strains were seeded separately on the same selective medium as controls. Some clones grown on the experimental plates were purified. To further confirm that they were true transconjugants, and not spontaneous KAN^R^ mutants of the donor, they were seeded on plates with NAL to corroborate their NAL^R^ phenotype. For transformation, the same *E. coli* K12 recipient strain was made competent using the standard protocol with calcium chloride (Sambrook et al., 1989). Transformants were selected on plates with trimethoprim.

### 2.4 DNA sequencing

All DNA sequencing was performed at Macrogen Inc. (Seoul, Korea). PCR-amplicons were sequenced by the Sanger method. Plasmids from five UPEC strains that had been transferred by conjugation to an *E. coli* K12 were extracted with the NucleoSpin Plasmid kit (Macherey-Nagel GmbH & Co. KG, Germany) and outsourced to Macrogen for sequencing. The library was prepared using a Nextera XT DNA Library Preparation kit (Illumina) and NGS was performed with an Illumina HiSeq X platform, obtaining paired-ended reads of 151 bp. Reads were quality-trimmed with Trimmomatic and cleaned with Bowtie2 to remove *E. coli* W3110 chromosome contaminating reads (GenBank: CP017979). The assembly was carried out with Unicycler within the BV-BRC server (https://www.bv-brc.org/) (Olson et al., 2023), and gene annotation was performed with Prokka 1.14.6 (Seemann, 2014).

### 2.5 GenBank accession numbers

Plasmid sequences have been submitted to GenBank under the following accession numbers: p31_CH_-1, draft assembly (contig of 91.664 nt) (PP566049); p61_CH_-1m, complete sequence of 6.799 nt (PP537156); p16_CH_, draft assembly (contig of 63.631 nt) (PP566048); p46_CH_-1, complete sequence of 73.233 nt (PP537157); and p47_CH_-1, complete sequence of 76.288 nt (PP566047).

## 3 Results

This study first concentrated on completing the characterization of the genetic basis of the TMP^R^ in the 101 SXT^R^ UPEC collection, i.e., in the remaining 47 strains for which we lacked this information. Whenever possible, we sought to identify the genes responsible for the TMP^R^ phenotype as well as their genetic context. The final goal was to gather previous and present results to reach a general view on the genetics of cotrimoxazole resistance in *E. coli* and also on the mobile genetic elements involved.

The search for genes conferring TMP^R^ first focused on strains containing class 1 or class 2 integrons, and then addressed the study of the remaining SXT^R^ strains.

### 3.1 Genetics of resistance to trimethoprim in SXT^*R*^ strains carrying class 1 and/or 2 integrons

A survey of class 1 and 2 integrons had previously been performed on the entire UPEC collection of 230 strains: Int1 was detected by the presence of the gene markers *intI1* and/or *sul1* in 66 isolates, and Int2 in 19 isolates by detecting the gene for its integrase *intI2*; four of them carried both integrons. This gave a total of 81 strains that were considered integron-positive, 74 of which were SXT^R^ (91%): 59 Int1^+^ only, 11 Int2^+^ only, 4 with both Int1^+^ and Int2^+^ (Poey and Laviña, 2014; de los Santos et al., 2021). In 57 Int1^+^ SXT^R^ strains, the variable region could be amplified with primers widely used by many authors and in 54 of them a *dfrA* variant was identified, thus accounting for the TMP^R^ phenotype (Figures 1B, 2) (Poey and Laviña, 2014, 2018; de los Santos et al., 2021).

From here on, the results correspond to the present work. We first analyzed the three Int1^+^ strains in which there was no *dfrA* gene in the variable region. One of them also contained an Int2, which provided a *dfrA1* gene (see below). The other two strains contained complex integrons, as an IS*CR1* was PCR-detected in them; due to the many different arrangements that can be found in such complex Int1 elements and that they usually contain *dfrA* genes of infrequent types, no further efforts were made to identify the genes responsible for the TMP^R^ phenotype of these two strains (Figures 1B, 2) (Toleman et al., 2006).

It still remained to identify the TMP^R^ determinants in six Int1^+^ strains whose variable region had not been able to be amplified. They all lacked *sul1*, indicating that they were deleted for all or part of the integron's 3' conserved region. Then, the primer pair F-RV and R-RV, adjusted to the *Pc*-variable region, was used, and in three cases an amplicon was generated and then sequenced. The three strains had the same gene content: a *Pc*_*W*_ promoter and *dfrA5* as a unique gene cassette (Figures 1B, 2). In the remaining three strains, the search continued using primer pairs internal to the *dfrA* genes and thus it was found that they contained a *dfrA14* variant. Then, its association with an Int1 platform was confirmed by amplifying with primers F-RV and dfrA14-R, and a *dfrA14* cassette preceded by a *Pc*_*H*1_ promoter was found (Figures 1B, 2).

In relation to the Int2^+^ strains, the *dfrA1* gene was searched for using a pair of primers internal to this gene. As expected, the 15 SXT^R^ strains carrying an Int2 were positive for *dfrA1*, including the four cases in which the Int2 coexisted with Int1. The linkage of *dfrA1* with the *intI2* gene was confirmed by PCR in all the Int2^+^ strains.

It was considered that the remaining 27 SXT^R^ strains, although being negative for the integron markers (*intI1, sul1*, and *intI2*), might still retain remnants of an integron with gene cassettes encoding resistance to TMP. It should be remembered that all of them owed their resistance to SMX to a *sul2* gene. First, the 27 strains were PCR-assayed for the Int1 variable region employing primers tightly adjusted to the *Pc*-gene cassettes region. There was a successful amplification in six of them and, indeed, the amplicons contained *dfrA* gene cassettes: five a *Pc*_*W*_ followed by *dfrA5*, as a unique gene, and one a similar content, but with *dfrA7*. Therefore, although these six strains had been cataloged as Int1^−^, they ultimately resulted to keep the central part of an Int1 (Figures 1B, 2).

In sum, with the exception of the two IS*CR1*-containing strains, the SXT^R^ phenotype could be completely explained by the presence of well-defined *dfrA* genes contained in the variable region of class 1 or 2 integrons in 78 strains.

### 3.2 Genetics of resistance to trimethoprim in strains without integrons

In this part of the work, we specifically focused on identifying the genes responsible for TMP^R^ in the remaining 21 SXT^R^ UPEC strains. PCR-reactions were assayed with primers internal to the *dfrA* genes, and in 16 of them a *dfrA* gene could be identified: *dfrA1* in one, *dfrA14* in another, and *dfrA8* in 14 (Figure 2). With this approach, the genetic context of the genes remained unknown. As to *dfrA8*, it is known that it is not related to an integron but to a compound transposon (Tn*5091*). In this structure, *dfrA8* appears together with another gene, *sbcD*, encoding a subunit of a DNA repair exonuclease, both genes being flanked by IS*26* elements (Sundström et al., 1995).

To gain more insight into the genetics of these 21 remaining SXT^R^ strains, conjugation experiments were designed to attempt to transfer their TMP^R^ determinants to a laboratory *E. coli* K12 strain. Due to the resistance profile of the UPEC strains, this approach could be applied to 20 of them. In five crossings, TMP^R^ transconjugants grew, so that the determinants of this phenotype were located in transferable plasmids of the corresponding five UPEC strains. Their *dfrA* genes had already been identified by internal primers (1 *dfrA1*, 1 *dfrA14*, and 3 *dfrA8*), but, as already mentioned, their genetic context remained unknown. The plasmid content of the five transconjugants was extracted: each of them had a large plasmid, but one also contained a small one. This latter was transformed into competent *E. coli* K12 cells and TMP^R^ clones grew, thus proving that the small plasmid, which was called p61_CH_-1m, was responsible for that phenotype and had been mobilized during conjugation by an accompanying large conjugative plasmid. The five plasmids, each carrying a *dfrA* gene, were then sequenced: p61_CH_-1m (*dfrA1*), p31_CH_-1 (*dfrA14*), and the remaining three, p16_CH_, p46_CH_-1 and p47_CH_-1 (*dfrA8*). Their names are the same as those of the UPEC strains, and in the case of p61_CH_-1m, the “m” was added to indicate that it was mobilizable.

**Figure 3:**
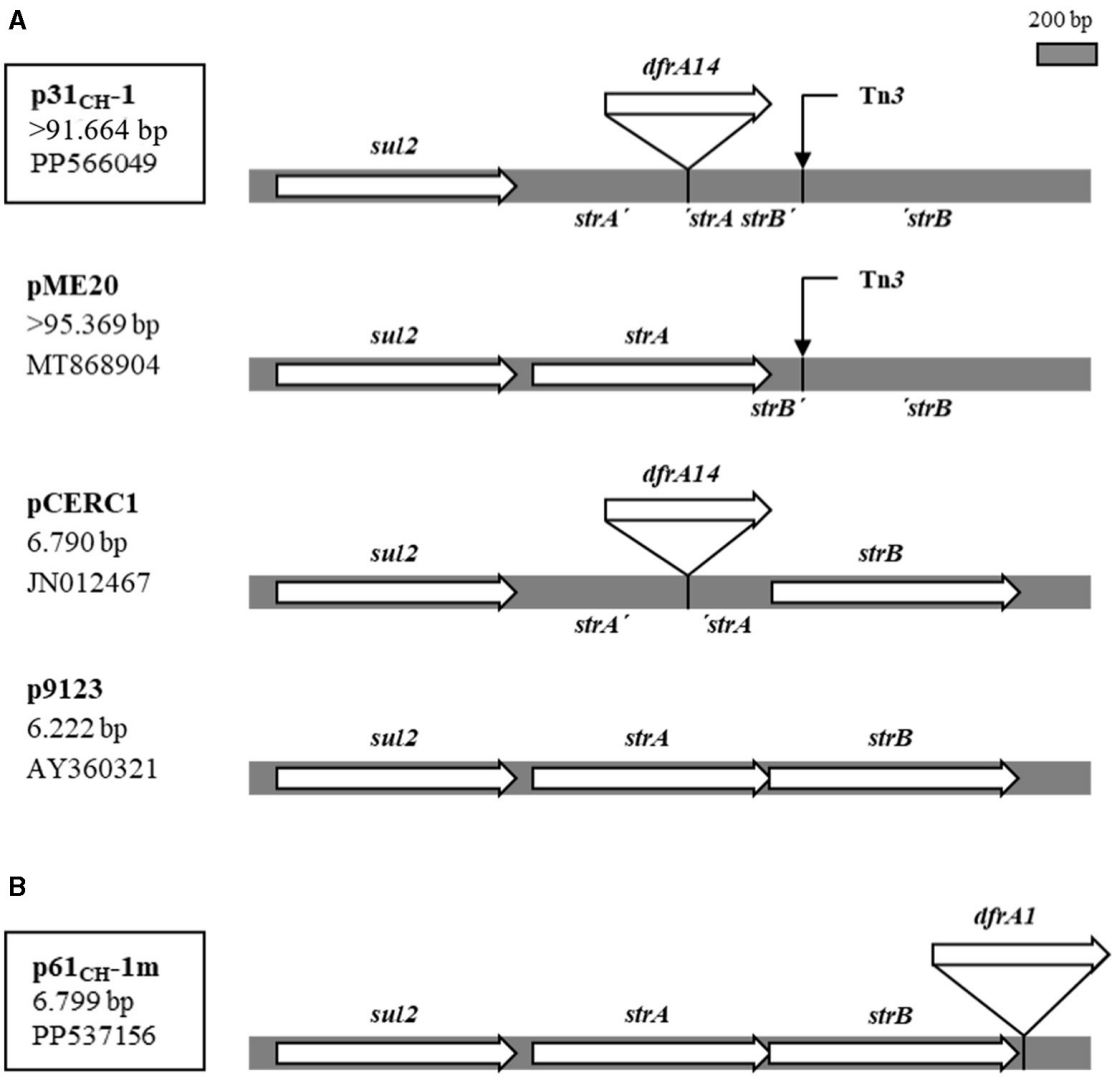
Single *dfrA* gene-cassettes inserted into the *sul2 strA strB* locus in plasmids p31_CH_-1 and p61_CH_-1m. **(A)** Structure of the locus in p31_CH_-1 compared with the same region in large and small plasmids with and without the *dfrA14* gene cassette insertion. The site of a Tn*3* insertion (4.957 bp) is indicated with a thin arrow. **(B)** The same locus with the *dfrA1* gene cassette insertion in p61_CH_-1m. At left, plasmids name, size and GenBank accession number. Plasmids from this work are boxed.

The *dfrA14* gene in the large conjugative plasmid p31_CH_-1 (>91 kb) was a single gene cassette that was located in a two-gene locus conferring streptomycin resistance, *strA strB*-also named *APH(3*′'*)-Ib* and *APH(6)-Id*, respectively- interrupting the *strA* gene. In fact, this same *strA'-dfrA14*-'*strA* structure had already been found in small, globally disseminated plasmids from strains of several bacterial genera, recovered in many countries. It was repeatedly found in the larger genetic cluster *sul2-strA'-dfrA14*-'*strA-strB* (Ojo et al., 2002; Anantham and Hall, 2012; Miranda et al., 2016). In the last years, more GenBank entries have appeared containing this gene arrangement and a few corresponded to large, possibly conjugative plasmids. The comparative analysis of this region of p31_CH_-1 with that of selected plasmids from the data bank is presented in Figure 3A. The comparison included a large plasmid, pME20 (from an *E. coli* isolated from a wastewater treatment plant in France), which presented the highest homology with p31_CH_-1 through its entire length but lacked the *dfrA14* cassette insertion, and two small plasmids, almost identical to each other except for the presence (pCERC1) or absence (p9123) of the *dfrA14* cassette. In addition, both large plasmids p31_CH_-1 and pME20 shared a transposon Tn*3* interrupting the *strB* gene exactly at the same position, a marker that points to their close relationship. Therefore, the *dfrA14* insertion into the *strA* gene of plasmid p31_CH_-1 was found to be widespread among small plasmids of several enterobacterial strains, always integrating the *sul2-strA'-dfrA14*-'*strA-strB* genetic arrangement, a locus that confers the phenotype of SXT resistance.

The mobilizable plasmid containing a *dfrA1* gene, p61_CH_-1m, was completely sequenced and found to be small, of 6.799 bp. The *dfrA1* gene was recognized as a single gene-cassette not linked to any integron. Instead, it was inserted 21 bp downstream of a *strB* gene, in a cluster *sul2-strA-strB-dfrA1* and being read in the same direction as the other genes in the arrangement (Figure 3B). In a Blastn search, four plasmids appeared with *dfrA1* in the same context: two of them, proceeding from *E. coli* isolates from racehorses in Hong Kong, were quasi-identical to p61P-1m, and the other two were larger plasmids from strains of the *Acinetobacter* genus isolated in China. Many other GenBank entries corresponded to plasmids very similar to p61P-1m, but all lacked the *dfrA1* gene. Therefore, plasmid 61_CH_-1m contained a novel gene arrangement conferring SXT resistance. Regarding its condition of mobilizable, a putative *oriT* sequence was identified in p61P-1m (gggtttcggggcgcagccctgaaccagtcatgtagcgctagcggagtgtatactggctta), which was of the ColE1 type (Francia et al., 2004).

The three remaining plasmids that were sequenced, p16_CH_, p46_CH_-1, and p47_CH_-1, were all conjugative and quite large. They were highly homologous to each other and all contained a *dfrA8* gene into the transposon Tn*5091*. The three plasmids had the same point of insertion of the transposon, close to a *bla*TEM gene, and quite near this latter there was a *sul2-strA-strB* locus. As to the remaining 11 strains with *dfrA8*, it seems reasonable to assume that they all belonged to the same transposon structure. Tn*5091* was first described in 1995 (Sundström et al., 1995) and since then *dfrA8* has been detected in many surveys performed in different parts of the world. When searching in the GenBank with Blastn, using the sequences of *dfrA8* and of Tn*5091* as queries, matches coincided, indicating that *dfrA8* is in fact a conserved constituent of this transposon. In addition, it was observed that Tn*5091* was generally located in large plasmids.

In our search for the genetic bases of the TMP^R^ in the 21 SXT^R^ strains considered here, we failed to find them in five strains. Three further possibilities were then assayed. First, considering that the five strains were sensitive to streptomycin and *sul*2^+^, we investigated the possible presence of an untested *dfrA* gene cassette insertion within a *strA* gene, as was the case in plasmid p31_CH_-1. The search was performed by PCR with primers annealing to the beginning and end of *strA* so as to detect if an insertion had occurred between them (Table 1). The result was that there was no amplification at all, unlike other strains from the collection that were *strA*^+^ (band of 857 bp) or to a strain with p31_CH_-1 (band of 1,425 bp). Other possible determinants of a TMP^R^ phenotype could be the rare *dfrB* genes (8 variants), which are Int1-associated gene cassettes; this was assayed with a generic pair of primers and no amplicon was generated (Table 1). Finally, we considered that a mutation in the chromosomal gene *folA*, encoding the dihydrofolate reductase of the Fol pathway, could be responsible for the TMP^R^ phenotype. Then, the *folA* gene and its upstream sequences were amplified and sequenced, but no changes appeared relative to the wild type genotype (Table 1).

**Figure 4:**
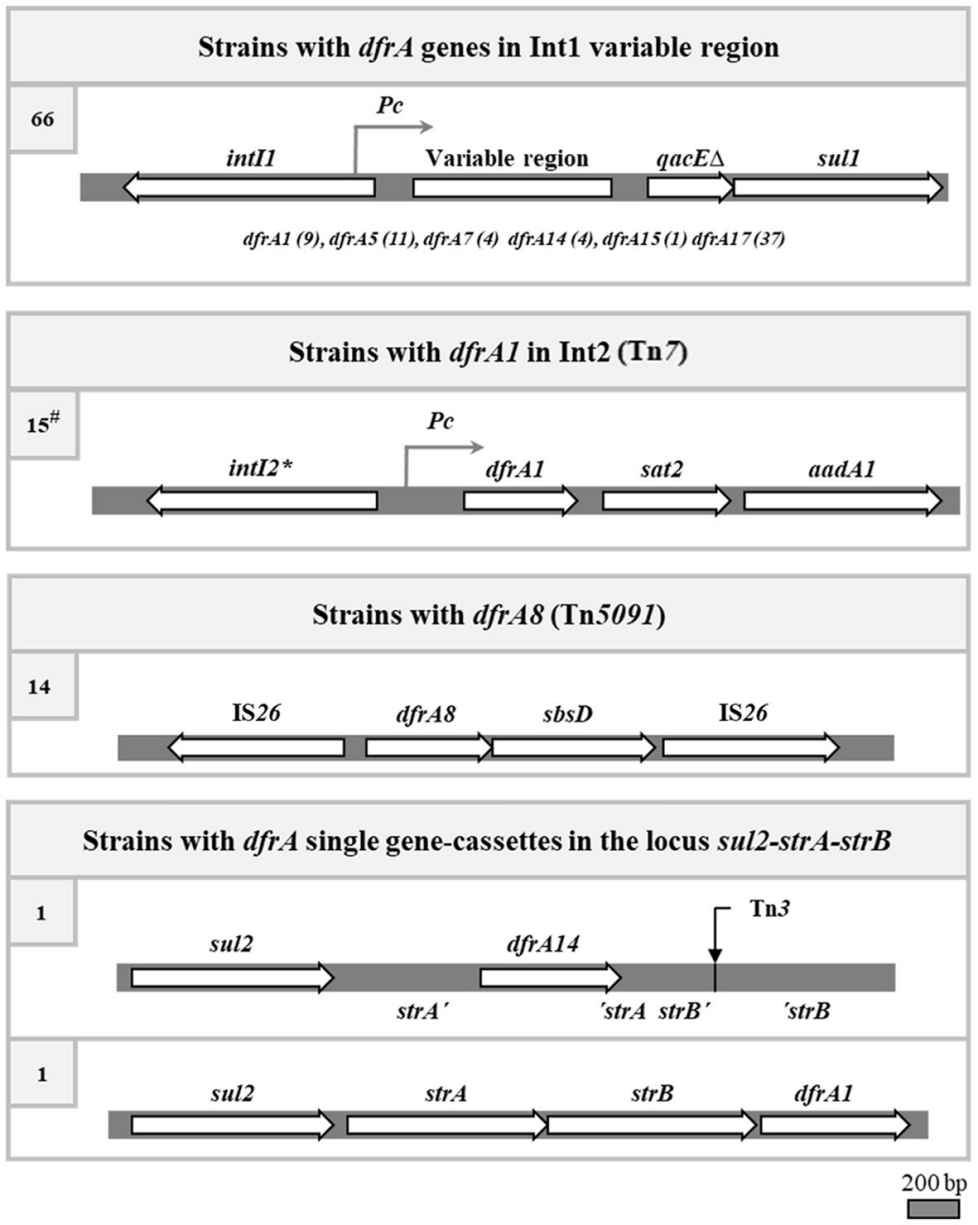
The four identified genetic contexts responsible for the trimethoprim resistance in the SXT^R^ strains. The genetic maps of the canonical integrons and transposons are depicted, although some strains may have deletions of parts of them. *, stop codon inactivating the *intI2* gene. At left, number of strains carrying each type of structure. #, four of the 15 strains with an Int2 also have an Int1, and in one of them the *dfrA1* gene of Int2 is the only responsible for the TMP^R^ of the strain.

In sum, considering previous and present results on the 101 SXT^R^ UPEC strains, it was found that 78 owed their phenotype of TMP^R^ to a *dfrA* gene cassette contained in a class 1 or 2 integron, two to *dfrA* single gene cassettes, and 14 to the *dfrA8* gene carried in a transposon (Figure 4). Curiously, no strain contained the *dfrA27* gene cassette nor the highly frequent *dfrA12* one.

## 4 Discussion

In this work we present the genetic bases of cotrimoxazole resistance in a collection of 101 uropathogenic *E. coli* strains. The study integrates previous and present results to provide the most complete understanding possible of the joint resistance to both antifolate components of the SXT formulation. In addition, considering the systematic strategy followed to collect the strains, the view provided here acquires epidemiological significance.

A generalized observation is that resistance to antifolates is strongly related to the presence of clinical integrons in many Gram-negative isolates from specimens of infected material. SXT is one of the treatments of choice for urinary tract infections, which are most frequently caused by *E. coli* strains, so testing for SXT resistance in UPEC isolates is routinely performed in the clinics. In a recent article, a 100% association was found between the phenotype of SXT resistance and the detection of class 1, 2, and 3 integrase genes when PCR reactions were applied directly to urine samples (Elias et al., 2022). In the present work we also found an important, albeit lower association, around 80%, between SXT^R^ and class 1 and 2 integrons. To reach this figure we had to survey many portions of integrons, particularly of Int1, in a significant number of UPEC isolates.

Int1 or remnants of it were thoroughly searched for and were found in 69 of the 101 SXT^R^ strains. In all but three, a *dfrA* gene cassette was found in the variable region, which explained the TMP^R^ phenotype of the strains (Figure 1). Although more than 40 *dfrA* variants have been described (Ambrose and Hall, 2021; Alcock et al., 2023), only six were found in the Int1 sequences of our SXT^R^ collection, and these were among the most frequently found in multiple surveys worldwide. As observed by other authors, the most commonly found cassette arrays were *dfrA17-aadA5* and *dfrA1-aadA1*, while *dfrA5* and *dfrA7* appeared as unique cassettes (Figure 1) (Domingues et al., 2015). One of the strains that lacked a *dfrA* gene in its Int1 variable region also had an Int2, which provided its *dfrA1* gene. The other two were positive for the ISC*R1* element, a marker of a complex Int1 structure that contains non-cassette resistance genes outside the Int1 variable region, *dfrA* among them. These latter *dfrA* genes are quite unusual and are specifically found in ISC*R1* complex integrons, e.g., *dfrA10, 11, 19*, among others (Toleman et al., 2006; Ambrose and Hall, 2021). It seems very likely that the two ISCR1^+^ strains in our collection possess one of these *dfrA* genes.

Two apparently abundant *dfrA* variants, as found in databases and in multiple reports, are *dfrA12* and *dfrA27*. However, these were not found in our collection, a result that raises the question of whether there could be differences in gene prevalence between geographic regions and time of strain collection. This topic has been addressed by some groups with varying results. In a survey of integrons and genes conferring SXT^R^ performed on UPEC strains from Europe and Canada in 1999 and 2000, no regional dependence was observed (Blahna et al., 2006). On the contrary, other authors found differences in the prevalence of *dfrA* variants and of gene cassette arrays in surveys conducted in other regions as well as in follow-up studies over several years (Yu et al., 2003; Su et al., 2006; Dahmen et al., 2010; Manyahi et al., 2017). Perhaps the *dfrA* set of variants found in our study may indeed reveal a certain bias with respect to other parts of the world and to the time period in which the UPEC isolates were collected (2007–2009).

An important focus in this work was on the Int1 central expression unit, composed of a *Pc* promoter and the gene cassettes under its transcriptional influence. A close relationship was found between these elements: the most frequent cassette array, *dfrA17-aadA5*, was invariably under the *Pc*_*H*1_ promoter, while *Pc*_*W*_ alone or reinforced by *P*_2_ generally preceded the other types of arrays (Figure 1). In fact, the number of different combinations of all these central elements was quite limited. The Int1 central expression unit analyzed here was found in canonical integrons, with their entire structure (40), but also in remnants lacking the *intI1* gene from the 5′ CS (15), or lacking *sul1* from the 3′ CS (8), or lacking both (6). Considering these results and our previous analyses on the whole UPEC collection, it should be noted that no strain was found with only the 5′ CS and only one just conserved the 3' CS (*qacE*Δ-*sul1*) (Poey and Laviña, 2014, 2018; de los Santos et al., 2021). This suggests that the Int1 variable region would paradoxically be the most “conserved region” of the integron, being particularly selected over time.

Gene cassettes are the mobile genetic elements that characterize integrons, and each is a gene followed by its cognate *attC* site (Patridge et al., 2009; Hall, 2012; Cury et al., 2016). In most cases, they can be simply recognized as such when located in the variable region of an integron, but they must be analyzed at the sequence level when they are outside their normal integration site. This is what happened with two of the SXT^R^ strains in our collection, whose *dfrA* genes could only be detected by PCR using primers internal to these genes, being one *dfrA14* and the other *dfrA1* (Figure 3). They were carried by plasmids that could be transferred by conjugation to an *E. coli* K12 and then were sequenced. Both *dfrA* genes proved to be true gene cassettes, each one with its specific *attC* site, but located outside an integron; that is, they were single gene cassettes.

The *dfrA14* cassette, carried by a large conjugative plasmid, was inserted within a *strA* gene. This same location has been found repeatedly in small plasmids from different bacterial genera in many countries, and the gene arrangement described is *sul2-strA'-dfrA14-'strA-strB*. The question arose as to how it had been transferred horizontally since it was carried by small plasmids that apparently could not be mobilized by conjugation (Ojo et al., 2002; Anantham and Hall, 2012; Miranda et al., 2016). However, the same insertion was also detected in *E. coli* isolates from food animals in Lithuania and could be transferred by conjugation, although the plasmid containing it was not further analyzed (Šeputiene et al., 2010). In the present work, a variant of the described arrangement was found in a conjugative plasmid, p31_CH_-1, which included the addition of a Tn*3* insertion in *strB*: *sul2-strA'-dfrA14-'strA-strB'-*Tn*3-'strB*. As shown in Figure 3A, small and large plasmids contain the region of reference, with or without the *dfrA14* insertion, but both large plasmids share the same Tn*3* insertion. In fact, p31_CH_-1 and pME20 are highly homologous to each other in all their extension, and their main difference is the presence or not of the *dfrA14* gene cassette into *strA*. Therefore, pME20 would be representative of a p31_CH_-1 precursor, and their comparison indicates that p31_CH_-1 could have acquired the *dfrA14* insertion by exchange of a DNA segment, for example with a small plasmid carrying the *sul2-strA'-dfrA14-'strA-strB* arrangement. This would most likely be operated by homologous recombination occurring in sequences flanking the *dfrA14* cassette in a situation in which both the large conjugative plasmid and a small plasmid with the cluster *sul2-strA'-dfrA14-'strA-strB* coexisted in the same bacterium. Then, the presence of the arrangement in conjugative plasmids explains its horizontal transfer between strains, species and related genera. Curiously, the UPEC carrying p31_CH_-1 was isolated from a child with urinary tract infection in 2008, a date that coincides with an outbreak of shigellosis in Chile caused by a strain of *Shigella sonnei* carrying the same *sul2-strA'-dfrA14-'strA-strB* arrangement in a small plasmid (Miranda et al., 2016). At this point, it should be recognized that this genetic arrangement is undoubtedly successful given its wide dissemination among bacteria all over the world, and would represent a gene combination different from integrons that confers resistance to cotrimoxazole.

As to the single *dfrA1* gene cassette in the small mobilizable plasmid p61_CH_-1m, it was found to be included in the arrangement *sul2-strA-strB-dfrA1*, being inserted very close to the end of *strB*, but not interrupting any gene (Figure 3B). Therefore, *dfrA1* must be expressed together with *strA* and *strB* as an operon. In this case, only four entries in GenBank presented this gene cluster and, notably, two plasmids from racehorses in Hong Kong (in releases dated 2022) were quasi-identical to p61_CH_-1m in extension and content, a fact that could be related to the export of this type of horses from our region to Hong Kong. Apparently, the genetic arrangement *sul2-strA-strB-dfrA1* in p61_CH_-1m has not been widely spread, so that it appears to be a relatively new gene arrangement conferring SXT resistance. In fact, it could be considered that this locus might have been formed in the very context in which the UPEC carrying this plasmid was isolated from the infected urine of a child. Although the isolate was collected some 15 years ago, it should be kept in mind that the horizontal transfer of the arrangement would depend on the chance of coexistence of the mobilizable small plasmid carrying it with a compatible conjugative one, and this requirement could reduce the probability of its spreading. In this sense, a putative ColE1 type *oriT* sequence was detected in p61_CH_-1m. This would make it susceptible to mobilization by conjugative plasmids belonging to several incompatibility groups. For example, these include members of the broad host range group IncP, which is known to spread antibiotic resistance genes among Gram-negative bacteria (Francia et al., 2004; Popowska and Krawczyk-Balska, 2013). Therefore, it could be expected that the wide dissemination of the *sul2-strA-strB-dfrA1* locus found in p61_CH_-1m is only a matter of time.

As to the 15 strains bearing an Int2, they all contributed to the SXT^R^ with a *dfrA1* gene, in accordance with their fixed structure. Therefore, summing up the 78 strains with *dfrA* genes inside class 1 and 2 integrons, the two strains with complex Int1 containing an ISCR*1*, plus two with the single gene cassettes *dfrA1* and *dfrA14*, there were still a good number missing to reach the total of 101 SXT^R^ strains. Of them, 14 resulted to have a *dfrA8* gene, known to be part of the composite transposon Tn*5091*, as corroborated in the three sequenced *dfrA8*-carrying plasmids. These also contained a *sul2* gene, which together with the *dfrA8* gene conferred the SXT^R^ phenotype. It should also be mentioned that in the three plasmids *sul2* was found in a locus *sul2-strA-strB*, a few kilobases apart from the Tn*5091*.

In sum, in this work we elucidated the genetic basis of TMP resistance in 40 of the 47 SXT^R^ strains of the collection that remained to be analyzed. Combining these results with previous findings, four main genetic contexts responsible for the TMP^R^ phenotype were identified: class 1 integrons, class 2 integrons, transposon Tn*5091*, and *dfrA* single gene-cassettes integrated into the *sul2-strA-strB* locus (Figure 4). These data were then correlated with the already known *sul* content of the strains, so that the genetic basis of the cotrimoxazole resistance were determined in 94 of the 101 SXT^R^ strains of the collection (Poey and Laviña, 2018; de los Santos et al., 2021). In these strains, the genetic linkage between the *sul* and *dfrA* genes varied depending on the genetic element involved. The loosest linkage would be that of the Int2 with *sul2*: Tn*7*, which contains the Int2, is most frequently located in the chromosome at its attachment site and we found no reference of its proximity to a *sul* gene (Peters, 2014). Similarly, *dfrA8*, contained in Tn*5091*, would not be closely linked to a *sul* gene, although the 14 strains carrying it were positive for *sul2*. The three plasmids *dfrA*8^+^ that were sequenced in this work contained the entire transposon and, quite near it, a *sul2* gene included in the *sul2 strA strB* locus; therefore, *sul2* and *dfrA8* were in the same replicon and had been co-transferred by conjugation. Class 1 integrons and the two *sul2 strA strB* derivative structures containing *dfrA* gene cassettes exhibited the most compact arrangements bringing together the two types of antifolate resistance genes. It could be thought that the evolutionary trend is toward the co-carriage of *sul* and *dfrA* genes in the same mobile genetic element so that they can be transferred together to new cellular hosts. The pressure exerted by the clinical use of cotrimoxazole should have strongly influenced in this direction. Since the use of sulfonamides preceded that of TMP, the insertion of *dfrA* genes close to a preexisting *sul*-containing genetic structure must have been selectively favored. The two most commonly found *sul* genes are *sul1*, which is part of class 1 integrons, and *sul2*, which is often found adjacent to the *strA strB* genes (Yau et al., 2010; de los Santos et al., 2021). These are precisely the most compact structures encoding SXT resistance.

Taking into account that antifolates are synthetic, resistance to them should have appeared in clinical isolates after the introduction of these drugs in antimicrobial treatments, i.e., after some 80 years ago for sulfonamides and 50 for TMP. In this sense, there is a collection of historical strains (1917–1954) -the Murray collection- of several hundred isolates of mainly Enterobacteriaceae, including many clinical species that are today enriched in antibiotic resistance genes. This collection is being analyzed by some groups in the world and, significantly, no traces of clinical integrons or of antifolate resistance genes have been found (Baker et al., 2015; Sütterlin et al., 2020). Therefore, it can be presumed that antifolate resistance is a relatively recent phenomenon, at least among clinical Gram-negative bacteria, and that it may still be actively evolving. In particular, the pressure exerted by the intensive administration of cotrimoxazole must have been strong enough to select for clones bearing both *sul* and *dfr* genes, which predominate over those carrying only one of these genes (de los Santos et al., 2021). As previously mentioned, *sul* genes integrate or accompany clinical integrons and *dfrA* genes are mostly located inside them, so that these mobile genetic elements could have reached their current structure and bacterial hosts in the last decades. For instance, *dfrA* genes are almost always in the first position in integron arrays, which indicates that these genes were the last to be acquired. It should be remembered that soon after the introduction of TMP into medical practice, descriptions appeared of enterobacterial clinical isolates, particularly from infected urines, that were highly resistant to TMP. In one of them, only 3 years after TMP became available for general use in medicine, R factors (resistance plasmids) encoding resistance to SXT were detected through transferring them to an *E. coli* K12 by conjugation (Fleming et al., 1972). Most probably, these plasmids carried already formed integrons.

In our collection, we found that class 1 integrons, endowed with the resource of a site-specific recombination system, were the main responsible for SXT^R^, but we also found that there were other elements contributing to this phenotype. Transposons, represented by Tn7, which contains an already fixed class 2 integron, and Tn*5091*, carrying the *dfrA8* gene, both equally conferred TMP resistance to a good number of additional SXT^R^ strains, while coexisting with an unlinked *sul2* gene. Two further genetic structures were detected, involving *dfrA* single gene cassettes that associated with a specific locus formed by the *sul2 strA strB* genes, which may represent the beginnings of well-structured new genetic elements encoding SXT resistance. The recent origin of both arrangements can be deduced from the very limited distribution of one of them (*sul2- strA-strB-dfrA1*), and from the persistence of the intact sequence of *strA*, turned into a pseudogene by the insertion of a *dfrA14* gene, in the other structure.

Finally, some of all these genetic structures conferring antifolate resistance could be horizontally transferred by conjugation, conjugative mobilization and even transduction, as shown previously (Poey and Laviña, 2018) and in the present communication. In sum, the idea emerges that the genetic structures conferring SXT resistance in Gram-negative bacteria are constantly evolving by recombination, transposition and horizontal gene transfer.

## Data availability statement

The datasets presented in this study can be found in online repositories. The data has been deposited at https://www.ncbi.nlm.nih.gov/ with accession numbers: PP566047–PP566049.

## Ethics statement

Ethical approval was not required for the study involving humans in accordance with the local legislation and institutional requirements. Written informed consent to participate in this study was not required from the participants or the participants' legal guardians/next of kin in accordance with the national legislation and the institutional requirements.

## Author contributions

MP: Conceptualization, Investigation, Methodology, Supervision, Writing – review & editing. ES: Conceptualization, Investigation, Methodology, Writing – review & editing. DA: Investigation, Writing – review & editing. CG-L: Data curation, Investigation, Methodology, Software, Writing – review & editing. ML: Writing – original draft, Writing – review & editing.
